# Workplace violence toward mental healthcare workers: A call for action 

**DOI:** 10.5249/jivr.v9i2.982

**Published:** 2017-07

**Authors:** Habibolah Khazaie, Alireza Ahmadi, Azad Maroufi

**Affiliations:** ^*a*^Sleep Disorders Research Center, Kermanshah University of Medical Sciences (KUMS), Kermanshah, Iran.; ^*b*^Pain & Injury Management Research Center, Kermanshah University of Medical Sciences (KUMS), Kermanshah, Iran.; ^*c*^Department of Psychiatry, Kurdistan University of Medical Sciences, Sanandaj, Iran.

Recently, one of the faculty members of Tabriz University of Medical Sciences was severely attacked in the hospital’s clinic by a patient with psychotic symptoms. Apart from the terrible and dramatic dimensions of the incident, this violent behavior can be carefully considered in several aspects as a Workplace Violence (WPV). First of all, this patient was a man with psychotic characteristics diagnosed with schizoaffective disorder and had included some of the staff in his delusional system. Second, the aim of this serious attack was a psychiatrist older than 40 with nearly 15 years of clinical and academic experience. This professor has specific experience in working and communicating with patients with various disorders. Therefore, although the patient had several patient-related risk factors for WPV, the psychiatrist who was attacked did not have any staff-related risk factors. Thus, this incident should be considered as a serious warning for the policy makers of psychiatry in the country and cause to arise several important questions.

WPV is defined as exerting intentional physical and psychological force and pressure on individuals to harm, threaten, or insult them in the workplace.^1^ Currently, WPV in the healthcare systems has become a major problem for the health and productivity of employees. ^2^The highest rates of WPV in healthcare systems have been reported in Emergency and Psychiatry wards. ^3^ Studies have shown that one out of every five patients admitted to acute psychiatric wards acts violently towards the staff.^4,5^ Moreover, most physical forms of WPV occur by patients in psychiatry wards; while in other medical wards verbal violence by those accompanying the patients is more prevalent.^6^

Various risk factors have been introduced to create WPV. Some of these factors such as male sex, schizophrenia, substance abuse, and history of violence, are related to the patients.^7^ Other factors are mostly related to the personnel and include weak relationship with patients, being less than 40 years old, little clinical experience, female sex, low educational status, less work experience, and high anxiety.^8^ Some risk factors can also be related to the safety climate dominating the workplace. It has been emphasized that the safety climate of the workplace is a very important factor and its improvement could maintain the wellbeing and safety of the personnel.^9^ Although various risk factors have been determined, some studies have shown that almost 70% of healthcare personnel cannot predict the occurrence of WPV.^10^

The main question is whether relying solely on the professional and personal skills of staff in mental healthcare personnel can have a preventive effect on WPV. Moreover, the unpredictability of such incidents makes the WPV far more out of control for the staff. The second question is whether measures have been taken to provide a safe work environment based on national standards; and whether the country’s healthcare system is equipped with comprehensive and effective guidelines for preventing WPV in critical situations. On the other hand, the social effects of these incidents on the general public and the reactions of the victim are yet among the issues requiring further investigation. Finally, does the destigmatization of mental diseases in modern psychiatry cause to neglect the potential threats imposed by these patients?

In conclusion, it could be stated that approaches towards destigmatization of psychiatric patients should not prevent us from searching for methods of controlling violence-related risk factors in the workplace. It seems that healthcare and educational centers in Iran need to implement security measures, and create safe environments, as well as compile comprehensive guidelines and protocols for protecting the wellbeing of their personnel. 

## References

[1] ILO, ICN, WHO, PSI. Framework Guidelines for Addressing Workplace Violence in the Health Sector 2002, http://www.who.int/violence_injury_prevention/violence/activities/workplace/en/, accessed 21 May 2014.

[2] Wu JC, Tung TH, Chen PY, Chen YL, Lin YW, Chen FL (2015). Determinants of workplace violence against clinical physicians in hospitals. J Occup Health.

[3] Arimatsu M, Wada K, Yoshikawa T, Oda  S, Taniguchi  H, Aizawa  Y (2008). An epidemiological study of work-related violence experienced by physicians who graduated from a medical school in Japan. J Occup Health.

[4] Ghoreishi A, Kabootvand S, Zangani E, Bazargan-Hejazi S, Ahmadi A, Khazaie H (2015). Prevalence and attributes of criminality in patients with schizophrenia. J Inj Violence Res.

[5] Iozzino L, Ferrari C, Large M, Nielssen O, de Girolamo G. Prevalence and risk factors of violence by psychiatric acute inpatients: a systematic review and meta-analysis. PLoS One. 2015; 10;10(6):e0128536. 10.1371/journal.pone.0128536 PMC4464653 26061796

[6] Carmi-Iluz T, Peleg R, Freud T, Shvartzman P (2005). Verbal and physical violence towards hospital and community-based physicians in the Negev: an observational study. BMC Health Serv Res.

[7] d’Ettorre G, Pellicani V. Workplace Violence Toward Mental Healthcare Workers Employed in Psychiatric Wards, Safety and Health at Work (2017). 2017: 1-6. 10.1016/j.shaw.2017.01.004 PMC5715456 29276631

[8] Zampieron A, Galeazzo M, Turra S, Buja A (2010). Perceived aggression towards nurses: study in two Italian health institutions. J Clin Nurs.

[9] Geiger-Brown J, Lipscomb J (2010). The health care work environment and adverse health and safety consequences for nurses. Annu Rev Nurs Res.

[10] Ferri P, Reggiani F, Di Lorenzo R. Aggressive behavior toward nursing staff in three different health care settings. [corrected]. Prof Inferm. 2011;64(3):143–150. Erratum in: Prof Inferm. 2014;67(3):192. 22044544

